# Circular RNAs are down-regulated in *KRAS* mutant colon cancer cells and can be transferred to exosomes

**DOI:** 10.1038/srep37982

**Published:** 2016-11-28

**Authors:** Yongchao Dou, Diana J. Cha, Jeffrey L. Franklin, James N. Higginbotham, Dennis K. Jeppesen, Alissa M. Weaver, Nripesh Prasad, Shawn Levy, Robert J. Coffey, James G. Patton, Bing Zhang

**Affiliations:** 1 Department of Biomedical Informatics, Vanderbilt University, Nashville, Tennessee 37232, USA; 2 Department of Biological Sciences, Vanderbilt University, Nashville, Tennessee 37232, USA; 3 Department of Cell and Developmental Biology, Vanderbilt University, Nashville, Tennessee 37232, USA; 4 Department of Medicine, Vanderbilt University, Nashville, Tennessee 37232, USA; 5 Department of Cancer Biology, Vanderbilt University Medical Center, Nashville, Tennessee 37232, USA; 6 Department of Pathology, Microbiology and Immunology, Vanderbilt University Medical Center, Nashville, Tennessee 37232, USA; 7 HudsonAlpha Institute for Biotechnology, Huntsville, Alabama 35806, USA

## Abstract

Recent studies have shown that circular RNAs (circRNAs) are abundant, widely expressed in mammals, and can display cell-type specific expression. However, how production of circRNAs is regulated and their precise biological function remains largely unknown. To study how circRNAs might be regulated during colorectal cancer progression, we used three isogenic colon cancer cell lines that differ only in *KRAS* mutation status. Cellular RNAs from the parental DLD-1 cells that contain both wild-type and G13D mutant *KRAS* alleles and isogenically-matched derivative cell lines, DKO-1 (mutant *KRAS* allele only) and DKs-8 (wild-type *KRAS* allele only) were analyzed using RNA-Seq. We developed a bioinformatics pipeline to identify and evaluate circRNA candidates from RNA-Seq data. Hundreds of high-quality circRNA candidates were identified in each cell line. Remarkably, circRNAs were significantly down-regulated at a global level in DLD-1 and DKO-1 cells compared to DKs-8 cells, indicating a widespread effect of mutant *KRAS* on circRNA abundance. This finding was confirmed in two independent colon cancer cell lines HCT116 (*KRAS* mutant) and HKe3 (*KRAS* WT). In all three cell lines, circRNAs were also found in secreted extracellular-vesicles, and circRNAs were more abundant in exosomes than cells. Our results suggest that circRNAs may serve as promising cancer biomarkers.

Circular RNAs (circRNAs) were first reported more than 30 years ago^1^,^2^,^3^,^4^, but had long been perceived as occasional RNA splicing errors until recent genome-wide analyses powered by next generation sequencing (NGS) technologies have shown these are bona fide RNA species. Studies during the past several years have identified a large number of exonic and intronic circRNAs across the eukaryotic lineage, including human, mouse, zebrafish, worms, fungi, and plants^5^,^6^,^7^,^8^. Based on the assumption that the abundance of circRNAs is much lower than that of linear RNAs, early studies typically use RNase R, a magnesium-dependent 3′ to 5′ exoribonuclease, to deplete linear RNAs before sequencing^9^. However, recent work showed that the abundance of circRNAs is similar to or higher than that of linear transcripts for about one in eight human genes^10^, which can be partially explained by higher cellular stability and longer half-life of circRNAs compared to linear mRNAs^11^. The observed high abundance of circRNAs suggests that RNase R treatment is likely to be unnecessary in NGS-based analysis of circRNAs, consistent with the identification of 7112 circRNA candidates from non-poly(A)-selected libraries generated by the ENCODE project^12^,^13^. It is now clear that circRNAs are evolutionarily conserved, exhibit cell-specific expression patterns, and are regulated independent of their linear transcripts^10^,^14^,^15^. For example, circRNAs are enriched in brain and accumulate to the highest levels in the aging central nervous system^16^,^17^. Recent studies also showed that circRNAs can be transferred to human exosomes^18^, where they are enriched and stable^19^. These findings suggest that circRNAs are prevalent, abundant, and potentially functional.

Knowledge about the general sequence features, biogenesis, and putative functions of circRNAs, especially exonic circRNAs, has gradually accumulated^11^. Because both circRNAs and linear RNAs are spliced from pre-mRNAs, the competition between circularization and linear splicing may play a role in the regulation of gene expression^20^. Moreover, introns between exons may be retained when exons are circularized^21^. Circularization of exonic circRNAs typically involves the canonical GU-AG splice site pairs^22^ and can contain one or multiple exons. On average, single-exon circRNAs form with exons that are three times longer than non-circularized exons^10^. Exon circularization is promoted by pairing of reverse complementary sequences within introns bracketing circRNAs; reverse complimentary sequences are primarily Alu repeats^23^,^24^,^25^. Two possible mechanisms for the formation of exonic circRNAs have been proposed, and both involve the canonical spliceosome^11^. Two circRNAs in mammals have been shown to function as miRNA sponges^5^, but significant enrichment of miRNA binding sites was not found for the majority of circRNA candidates^12^,^13^.

Although other non-coding RNAs have been shown to play critical roles in cancer, the association between circRNAs and cancer is largely unknown^26^,^27^,^28^. In this study, we performed deep RNA-Seq analysis of rRNA-depleted total RNA libraries to characterize circRNA expression in three isogenically-matched human colon cancer cell lines that differ only in the mutation status of the *KRAS* oncogene. The parental DLD-1 cells contain both wild-type and G13D mutant *KRAS* alleles, whereas the isogenically-matched derivative cell lines DKO-1 and DKs-8 contain only a mutant *KRAS* and a wild-type *KRAS* allele, respectively. *KRAS* mutations occur in approximately 34–45% of colon cancers^29^,^30^ and have been associated with a wide range of tumor-promoting effects^31^. We developed an integrated bioinformatics pipeline to identify, confirm and annotate circRNAs based on RNA-Seq data. Using the pipeline, we studied both cellular and exosomal circRNAs in the three cell lines, with confirmation of altered circRNAs in a second set of isogenically matched cell lines. To our knowledge, this is the first report describing the impact of a well-established oncogene on the abundance of circRNAs.

## Results

### Bioinformatics pipeline

**Figure 1:**
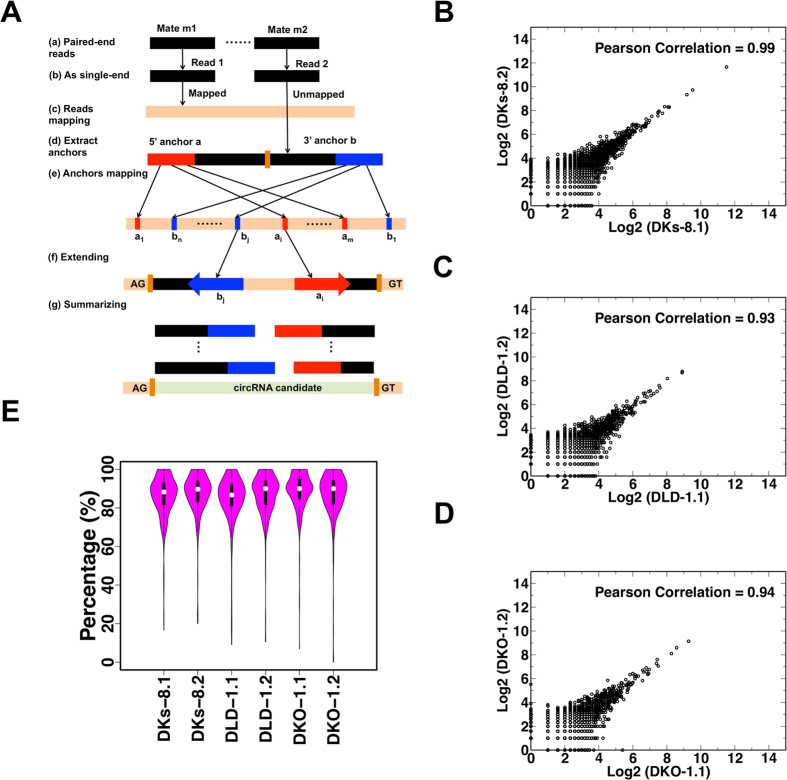
Bioinformatics pipeline and analysis of cell circRNAs. (**A**) Computational pipeline. (**B**–**D**) Pearson Correlation analysis between cell replicates for DKs-8, DLD-1, and DKO-1 colorectal cell lines, respectively. (**E**) Distribution of percentages of back-splice mates with corresponding mates that can be mapped (PCMM) for each sample.

Exonic circRNAs largely result from back-spliced exons, in which splice junctions are formed by an upstream 5′ splice acceptor and a downstream 3′ splice donor. Back-splice reads mapping to such junctions are the most important indicator for circRNAs that can be gleaned from RNA-Seq data^5^,^11^,^16^,^23^,^32^,^33^. Similar to the existing pipeline used by Memczak *et al*.^5^, our pipeline (Fig. 1A) uses the presence of back-splice reads to identify exonic circRNA candidates. However, multiple mapping positions are allowed when mapping anchors in our pipeline. Find-circ only reports a random mapping position and may therefore miss some circRNAs (false negatives). Moreover, because one read may be considered as a back-splicing candidate at one position or a linear gapped mapping at another position, find-circ may also introduce false positives. Thus, allowing multiple mapping position in our pipeline may help reduce both false positives and false negatives. Briefly, one paired-end read was used as two single-end reads for mapping to the genome. Mappable reads were discarded because back-splice reads cannot be mapped to the genome directly. The 5′ and 3′ termini of unmapped reads were then extracted as anchors, which were aligned to the genome independently with multiple mapping allowed. Because multiple mapping is allowed, all possible pairs of anchor alignments were evaluated. If any of these pairs correspond to a normal linear gapped mapping, the read was discarded. For the remaining reads, all the possible extensions that could be extended to reconstruct the original read with a maximum of two mismatches were further considered. Then we will search the GU/AC splice sites for each extension. If any extensions with the GU/AC splice sites, the read was considered as with GU/AC splice sites. Extended alignments flanked by GU/AG splice sites were used to define a back-splice read.

Contamination from other biological sources may affect both the identification and quantification of circRNAs. To check possible contaminations from bacteria and viruses, we built a database with all bacterial and viral sequences and blasted all back-splicing mates against the database. For cellular RNAs, 99.6% to 99.8% of the mates had no hits to the database and none of them had a hit with two or less mismatches. For exosomal RNAs, 91.8% to 99.4% of the mates had no hits to the database and only a few had a hit with two or less mismatches (Table S1). Next, all back-splicing mates were mapped to the bovine genome^34^ both linearly and using the back-splicing detection algorithm. The linear mapping percentages were close to 0 for all samples, and no more than 2.2% of the back-splicing mates could be back-splicing-mapped to the bovine genome (Table S2). These results show that the vast majority of the identified circular RNAs are not from bacterial and viral contamination and the potential contamination from the bovine sources is very limited. We discarded all back-splicing reads that can be mapped to bacterial, viral, or bovine genomes from downstream analysis to avoid any influence from possible contamination.

Sequence fragments supported by two or more remaining back-splice reads were considered as circRNA candidates, and those supported by ten or more back-splice reads were considered as high quality candidates. Finally, circRNA candidates with sequence fragment lengths between 100 and 1000,000 bp were reported by the pipeline.

### Identification of circRNA candidates in colorectal cancer cells

We prepared cellular RNA libraries from the three isogenic-*KRAS* CRC cell lines, each with two biological replicates. RNase R treatment was not applied during library construction. Sequencing was performed at high depth, with ~100 million reads per sample. Applying the above-described pipeline to cellular RNAs from the three cell lines identified thousands of circRNA candidates (Table S3) and hundreds of high quality circRNA candidates in each biological replicate (Table 1). Among the 1620 high quality candidates detected in our study, 1395 (86.1%) were found in the circBase database^35^.

To assess the reproducibility of the data, we generated scatter plots comparing the back-splice read counts of individual circRNAs from replicates of the three cell lines (Fig. 1B–D). As shown, the vast majority of all candidates were supported by consistent identification of back-splice reads in all replicates. Person’s correlations between replicates were 0.99, 0.93, and 0.94 for DKs-8, DLD-1, and DKO-1, respectively. These scatter plots show circRNA candidates with higher read counts are closer to the diagonal, suggesting that reproducibility tended to be higher for circRNA candidates with high back-splice read counts. Therefore, our downstream analyses focused only on high quality candidates with at least ten back-splice reads.

To further evaluate the reliability of the identified circRNA candidates, we leveraged the paired end information. As shown in Figure S1, if one mate of a paired end read mapped to a back-splice junction (Mate a), the corresponding mate could be mapped to the candidate circRNA sequence either within the circle (Mate b’) or crossing the back-splice junction (Mate b). For each high quality candidate, we calculated the Percentage of back-splice mates with Corresponding Mates that can be mapped to the candidate circRNA sequence (PCMM), *i.e.* the percentage of properly paired back-splice mates. As shown in Fig. 1E, the median percentages ranged from 88.2% to 90.0% across the six samples, suggesting high reliability of these circRNA candidates. We also tested RNase R resistance of circRNAs in the DKO-1 and DKs-8 cell lines with the top four most abundant circRNAs. As shown in Figure S2, these circRNAs were enriched by RNase R (R+) treatment compared to mock treated controls (R−). Thus circRNAs are resisted to RNase R treatment. Taken together, these results suggest that a large number of circRNAs can be reliably and reproducibly identified and quantified in the three cell lines.

### Down-regulation of circRNAs in *KRAS* mutant cells

**Figure 2:**
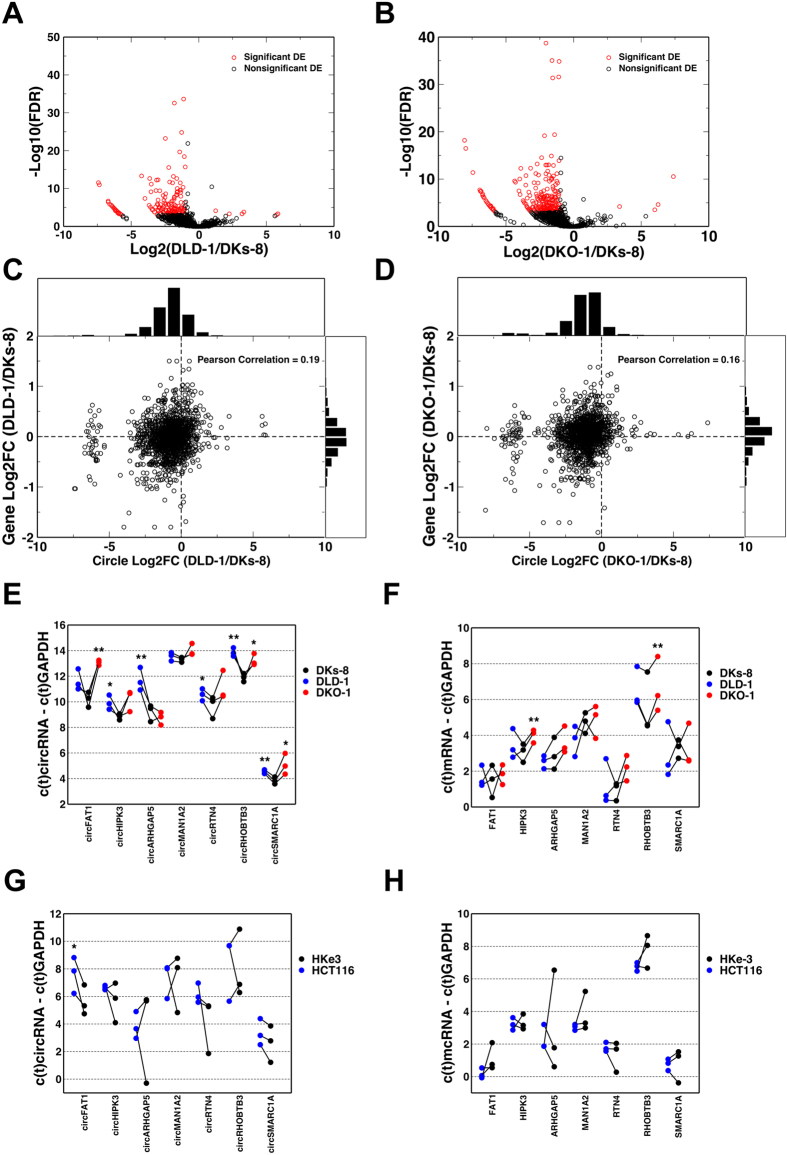
Differential expression analysis for cellular circRNAs. (**A**,**B**) circRNA differential expression analysis between mutant and wild-type *KRAS* cells. (**C**,**D**) Differential expression results for circRNAs and their host genes. Histograms of each gene and corresponding circRNAs log2FCs are shown above the X and Y-axes, respectively. (**E**) qRT-PCR results for seven selected circRNAs between mutant and wild-type cells. (**F**) qRT-PCR results for host genes of selected circRNAs. (**G**) qRT-PCR results for seven selected circRNAs between HCT116 and HKe3 cells. (**H**) qRT-PCR results for host genes between HCT116 and HKe3 cells. (Two-tailed, paired t-test was used for the analysis. *denote p values ≤ 0.1 and **≤0.05).

To test whether the expression levels of circRNAs are regulated by *KRAS*, we compared the levels of circular RNA candidates between the mutant and wild-type *KRAS* cell lines. circRNAs were globally down-regulated in the mutant *KRAS* DKO-1 (Fig. 2A) and DLD-1 (Fig. 2B) cell lines compared to the wild-type *KRAS* DKs-8 cell line. Specifically, 443 and 305 circRNAs were significantly down-regulated in DKO-1 and DLD-1 cells, respectively (False Discovery Rate [FDR] < 0.01 and Fold Change [FC] >2). In contrast, only 5 and 13 circRNAs were significantly up-regulated in DKO-1 and DLD-1 cells, respectively. Among the top ten most abundant circRNAs in distinct genes, seven were significantly down-regulated in DKO-1 cells and five of them were also significantly down-regulated in DLD-1 cells (Table 2). These results suggest that circRNAs are down-regulated in *KRAS* mutant cells at a global level.

We next sought to determine whether circRNA down-regulation was due to down-regulation of corresponding host genes. Figure 2C and D provide a direct comparison of the differential expression results for circRNAs and their host genes between each of the two mutant cell lines and the wild-type cell line. While the log-fold changes of the host genes exhibited a symmetrical distribution around 0, the log-fold changes of circRNAs were negatively shifted toward decreased abundance in mutant *KRAS* cell lines. The correlations between log-fold changes of circRNAs and host genes were 0.19 and 0.16, respectively, for the two comparisons. Using the most abundant circRNA candidate circRNA chr4:187627717-187630999 as an example, we found that this circRNA was down regulated by 6.6- and 5.3-fold in DLD-1 and DKO-1 cells, respectively compared to DKs-8 cells. In contrast, the host gene FAT1 was only down-regulated by 1.7- and 1.8-fold, respectively. These data suggest that circRNAs can be regulated independently of their corresponding host genes.

To validate our findings, we performed qRT-PCR analysis for seven out of the ten most abundant circRNA candidates. As shown in Fig. 2E, all were confirmed by qRT-PCR and six out of the seven circRNA candidates were significantly down-regulated in at least one mutant cell line compared with the wild-type cell line (two-tailed, paired t-tests was used for the analysis, where *are p values ≤ 0.1 and **≤0.05). As a comparison, Fig. 2F shows different trends for the host genes of these circRNAs. These results further confirm our finding that circRNAs are down-regulated in mutant *KRAS* cells and that the regulation of circRNAs can occur independent of their host genes.

To further strengthen our conclusion, we performed additional experiments using another pair of isogenically-matched human colon cancer cell lines, HCT116 and HKe3. Derived from a completely different cancer, HCT116 harbors mutant G13D *KRAS* while its clonal derivative HKe3 contains wild-type *KRAS*^36^. Consistent with our previous results, all seven circRNAs assayed were down regulated in the HCT116 cells compared to the HKe3 cell line as shown in Fig. 2G. Among them, circFAT1 was significantly down-regulated in the mutant *KRAS* cell line (HCT116). Furthermore, the host genes for these candidates were not significantly differentially expressed between HCT116 and HKe3 cell lines (Fig. 2H). These results support our finding that circRNAs are down-regulated in mutant *KRAS* cells and that the regulation of circRNAs can occur independently of their host genes.

### circRNAs in exosomes

**Figure 3:**
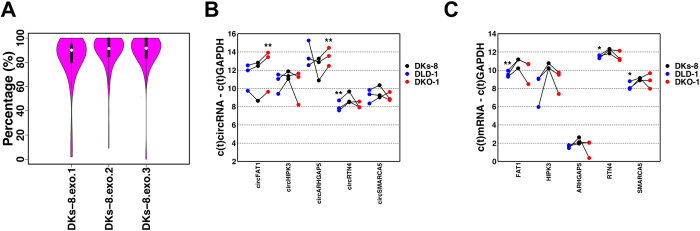
Bioinformatics analysis of circRNAs in exosomes. (**A**) Distribution of PCMM values for three DKs-8 exosomes replicates. (**B**) RT-PCR results for five selected circRNAs between mutant and wild-type cell lines in exosomes. (**C**) RT-PCR results for host genes of confirmed circRNAs in exosome. (Two-tailed, paired t-test was used for the analysis. *Denote p values ≤ 0.1 and **≤0.05).

Several recent reports have identified extracellular circRNAs^18^,^19^. To test whether circRNAs could be detected in the exosomes of colon cancer cell lines, we performed RNA-Seq analysis for exosomal RNAs from the three cell lines, each with three biological replicates. High quality circRNA candidates were identified in all three cell lines (Table S4). However, the number of high quality candidates varied among the replicates. Because the variation between DKs-8 exosomal replicates was relatively low, we focused our downstream analyses on data from DKs-8 derived exosomes. High quality exosomal circRNA candidates identified in this cell line were well supported by paired end information. Specifically, the median percentages of properly paired back-splice mates were 90.0%, 91.3%, and 91.7% for the three replicates, respectively (Fig. 3A). Table 3 shows the ten most abundant exosomal circRNA candidates in distinct genes in DKs-8 cells. Interestingly, seven of these circRNAs were also the top ten most abundant circRNAs candidates in DKs-8 cells (Table 2).

To validate the RNA-Seq results, qRT-PCR analysis was also performed on these consistently present and abundant circRNA candidates in exosomes. As shown in Fig. 3B, five of these circRNAs were confirmed as present in exosomes and three of them were differentially expressed in at least one set of mutant cell line derived exosomes compared with the wild-type cell line exosomes (two-tailed, paired t-tests was used for the analysis, where *are p values ≤ 0.1 and **≤0.05). Among them, circFAT1 was significantly down regulated in DKO-1 as compared to DKs-8 exosomes (Fig. 3B); this circRNA followed the same trend in cells (Fig. 2E). Meanwhile, circRTN4 was significantly up regulated in DLD-1 exosomes (Fig. 3B), while it was significantly down regulated in DLD-1 cells (Fig. 2E). The mRNA expression levels of these circRNA host genes were also tested by qRT-PCR and the results shown in Fig. 3C. The mRNA expression levels of both FAT1 and RTN4 were up regulated in exosomes from mutant *KRAS* cells. Therefore the shift in the relative circRNA levels was not the same as that for their linear mRNA host genes when comparing mutant and wild-type *KRAS* derived exosomes. These results suggested that there is a complex exosomal trafficking mechanism for circular RNAs. This is interesting given the increased abundance of RNA-binding proteins present in wild-type *KRAS* as compared to mutant *KRAS* cell-derived exosomes^37^. Results from the proteomic analysis of these exosomes may explain both the relative differences in circRNA and linear RNA content in DKs-8 as compared to DKO-1 and DLD-1 exosomes, as well as the relatively consistent levels of such RNAs in DKs-8 exosomes, given that such RNAs might be trafficked by these specifically exosomally-localized DKs-8 enriched RNA-binding proteins.

### Relative abundance of circular and linear transcripts

Because RNAse R treatment was not applied during the RNA library construction in this study, the resulting RNA-Seq data allowed us to directly compare the abundance of circRNAs and their linear host RNAs. Similar to previous studies^15^,^17^, we used the ratio between Expression level of exons With Circular RNAs and Expression level of exons with No Circular RNAs (EWC/ENC) to quantify the relative abundance of these two types of transcripts.

**Figure 4:**
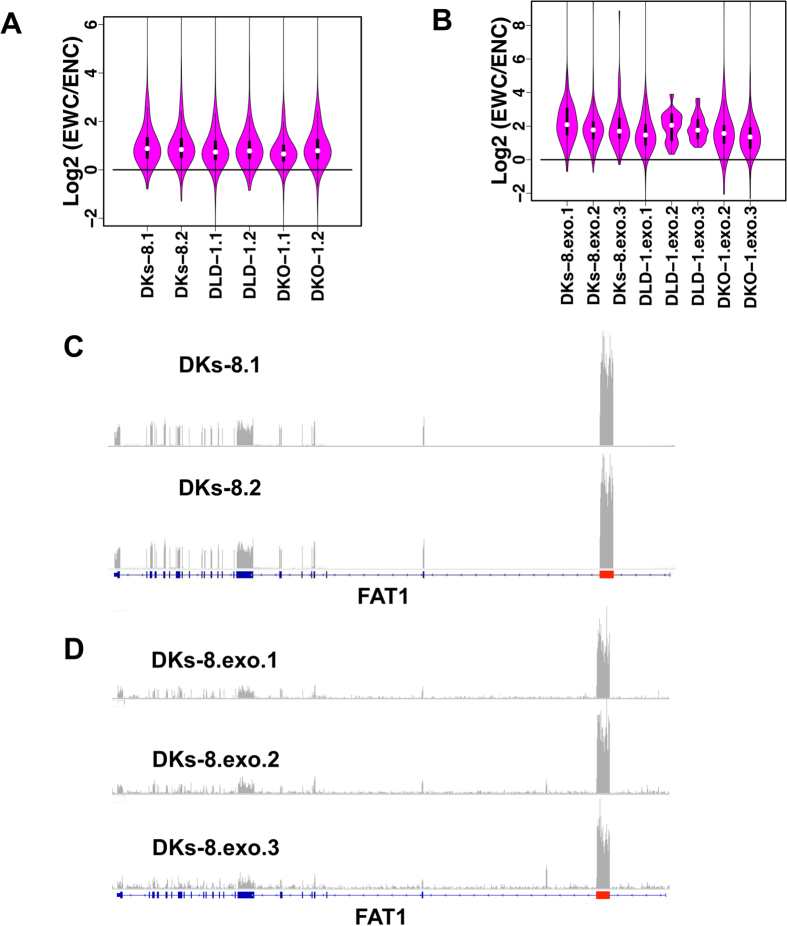
Relative expression levels of circRNAs compared to linear transcripts. (**A**,**B**) Distribution of EWC/ENC values for cellular and exosomal RNAs, respectively. DKO-1.exo.1 was excluded because only 2 high quality candidates were identified in this replicate (Table S4). (**C**,**D**) Expression levels of exons with and without circRNA with the FAT1 gene in DKs-8 cells and exosomes, respectively.

For cellular RNAs, the median EWC/ENC ratios ranged from 1.57 to 1.84 across the three cell lines (Fig. 4A). Similar analysis was performed on exosomal RNAs, where the median EWC/ENC ratios were much higher and ranged from 2.56 to 4.26 (Fig. 4B). Figure 4C and D show the read coverage depth plots for the most abundant circRNA circFAT1 (chr4:187627717-187630999) in DKs-8 cells and exosomes, respectively. The exon corresponding to circFAT1 (red) had a much higher read depth compared with other exons (blue). The EWC/ENC ratios were 3.5 and 3.0 for the two cell replicates, respectively, and 7.1, 7.6, and 7.7 for the three exosome replicates, respectively. These results are consistent with recent reports that circRNAs are more abundant than their host linear RNAs^15^,^17^ and provide additional evidence that circRNAs are likely to be more stable than their linear transcripts^10^,^11^. In addition, our results suggest that cirRNAs are enriched in exosomes, which is consistent with a recent publication^19^.

## Discussion

In this work, we determined the circRNA expression profiles in both cells and exosomes from three CRC cell lines that differ only in *KRAS* mutation status. Hundreds of high quality circRNA candidates were identified in cellular RNAs and we discovered that they could be transferred to exosomes. circRNAs tended to be more abundant in exosomes. Importantly, we showed that circRNA abundance was down-regulated at a global level in mutant *KRAS* cell lines, suggesting a potential involvement of circRNAs in oncogenesis.

There are complex regulatory mechanisms for both circRNA and host gene expression. Although circRNAs were down-regulated in both DLD-1 and HCT166 based cell lines, it is difficult to conclude that the circRNAs are directly regulated by *KRAS*. One possibility is that down-regulation of circRNAs in *KRAS* mutant cells is caused by their increased exporting to exosomes. However, as shown in Fig. 4B, the EWC/ENC median values were 2.77, 4.15, 3.38, 2.96 and 2.56 for *KRAS* mutant exosomes and were 4.26, 3.43 and 3.25 for *KRAS* WT exosomes (Fig. 4B). The median values in *KRAS* mutant exosomes were comparable to that in *KRAS* WT exosomes. Moreover, Fig. 3B shows that two of three significantly regulated circRNAs between *KRAS* mutant and WT exosomes were down-regulated in *KRAS* mutant (circFAT1 and circARHGAP5). These data suggest that circRNAs are not enriched in exosomes of the *KRAS* mutant cells. We also examined the expression levels of the RNA-editing enzymes *ADAR* and the RNA-binding protein QKI, which were reported as circRNA regulators^25^,^28^. The *ADAR* was decreased in the *KRAS* mutant cells, which may lead to an increase of circRNAs. QKI was down-regulated in *KRAS* mutant cells, which may lead to down-regulation of circRNAs. More broadly, we studied the expression levels of all RNA-binding proteins from RBPDB^38^, following the approach taken by Conn *et al*.^28^. Six were found to be differentially expressed (FDR < 0.01 and absolute log2FC > 1) in *KRAS* mutant cell lines compared with wild-type cell lines (*ELAVL2, RBMS3, BICC1, MSI1, RBM44, and LARP6*). Three of these were up regulated and the others were down regulated. The most up-regulated gene, *ELAVL2*, can function as an alternative pre-mRNA splicing regulator in mammalian neurons^39^,^40^. The most down-regulated gene, *MSI1*, is also an important post-transcriptional regulator^41^,^42^. These genes may serve as candidate circRNA regulators. However, our previous work shows that the correlation of mRNA and protein expression level is low for RNA-binding proteins^43^. Further investigation will be needed to precisely define how circRNAs are regulated.

## Methods

### Cell Culture

Cells were cultured in DMEM supplemented with 10% bovine growth serum until 80% confluent. To collect exosomes, cells were then washed 3 times with PBS and cultured for 24 hr in serum-free medium. The medium was collected and replaced with ionomycin-containing media for 1 hr, after which ionomycin-containing media was collected and pooled with the previously collected serum-free medium.

### Exosome isolation

Exosomes were isolated from conditioned medium of DKO-1, Dks-8, and DLD-1 cells, with slight modification^44^. Pooled media as describted above was centrifuged for 10 min at 300 × g to remove cellular debris, and the resulting supernatant was then filtered through a 0.22-um polyethersulfone filter (Nalgene, Rochester, NY) to reduce microparticle contamination. The filtrate was concentrated ~300-fold with a 100,000 molecular-weight cutoff centrifugal concentrator (Millipore). The concentrate was then subjected to high-speed centrifugation at 150,000 × g for 2 hr. The resulting exosome-enriched pellet was resuspended in PBS containing 25 mM HEPES (pH 7.2) and washed by centrifuging again at 150,000 × g for 3 hr. The wash steps were repeated a minimum of 3 times until no trace of phenol-red was detected. The resulting pellet was resuspended in PBS containing 25 mM HEPES (pH 7.2) and protein concentrations were determined with a MicroBCA kit (Pierce). The number of exosomes per ug of protein was determined by nanoparticle tracking analysis (NanoSight, Wiltshire, UK) and the results can be found in a recent publication from us in which the same exosome preparations were used (Figure S1A in that paper)^37^. Analysis was performed on three independent preparations of exosomes.

### RNA purification

Total RNA from exosomes and cells was isolated using TRIzol (Life Technologies). In the case of exosomal RNA isolation TRIzol was incubated with 100 ul or less of concentrated exosomes for an extended 15 min incubation prior to chloroform extraction. RNA pellets were resuspended in 60 μl of RNase-free water and were then re-purified using the miRNeasy kit (QIAGEN). Final RNAs were eluted with two rounds of 30 ul water extraction.

### mRNA library preparation and sequencing

Total RNA containing both long RNA as well as miRNA fractions was extracted from exosomes or cell lines using Trizol followed by miRNeasy Kit purification. Final elution was in 60 μl RNase free sterile distilled water. The concentration and integrity of the extracted total RNA was estimated by Qubit^®^ 2.0 Fluorometer (Invitrogen, Carlsbad, California), and Agilent 2100 Bioanalyzer (Applied Biosystems, Carlsbad, CA), respectively. RNA samples with a RIN value of at least 7.0 or higher were used for further processing.

Approximately 500 ng of total RNA was required for proceeding to downstream RNA-seq applications. Briefly, a Ribo-zero Magnetic Gold rRNA removal kit (Epicenter, IIlumina Inc.) was used to remove ribosomal RNA from the total RNA. Next, first strand synthesis was performed using NEBNext RNA first strand synthesis module (New England BioLabs Inc., Ipswich, MA, USA). Immediately, directional second strand synthesis was performed using NEBNExt Ultra Directional second strand synthesis kit. Following this, cDNAs were used for standard library preparation protocol using NEBNext^®^ DNA Library Prep Master Mix Set for Illumina^®^ with slight modifications. Briefly, end-repair was performed followed by polyA addition and custom adapter ligation. Post-ligated materials were individually barcoded with unique in-house genomics service lab (GSL) primers. Library quality was assessed by Qubit 2.0 Fluorometer, and the library concentration was estimated by utilizing a DNA 1000 chip on an Agilent 2100 Bioanalyzer. Accurate quantification for sequencing applications was determined using the qPCR-based KAPA Biosystems Library Quantification kit (Kapa Biosystems, Inc., Woburn, MA). Each library was diluted to a final concentration of 12.5 nM and pooled equimolar prior to clustering. Paired-End (PE) sequencing was performed on all samples. Raw reads were de-multiplexed using a bcl2fastq conversion software v1.8.3 (Illumina, Inc.) with default settings.

### circRNA identification

Reads with length 100 bp were mapped to the UCSC hg19 human genome (with mitochondrial sequences) by Bowtie 2 with up to 2 mismatches (version 2.2.3)^45^. Paired 3′ and 5′ end anchors with length 20 bp were extracted for each unmapped read. Anchor pairs were mapped to the above genome with no mismatches and up to 40 mapping positions using Bowtie 2. Refseq gene annotations from UCSC were used to annotate circRNA candidates^46^. Custom PERL scripts were used to implement the pipeline (Fig. 1A).

### Contamination analysis

We built a database with all bacterial and viral sequences from the NCBI nt database^47^ (ftp://ftp.ncbi.nlm.nih.gov/genomes/refseq/bacteria/assembly_summary.txt and ftp://ftp.ncbi.nih.gov/genomes/Viruses/). All back-splicing mates were blasted^48^ (ncbi-blast-2.3.0+) against the database with default parameters. Next, back-splicing mates were linearly mapped to the bovine genome^34^ by Tophat2 (version 2.0.12) with up to 2 mismatches^49^. Moreover, these mates were mapped to the bovine genome using the back-splicing detection algorithm described above.

### Differential expression analysis

To count reads mapped normally to genes, paired end reads were mapped to the hg19 human genome using Tophat2 with up to 2 mismatches. Htseq-count (version 0.6.0) with default parameters was used to count reads mapped to genes with the refSeq annotation^50^. It is worth noting that host gene expression was quantified using reads from both linear and circRNAs because existing tools cannot separate linear and circRNA counts based on RNA-Seq data from total RNAs. Accordingly, our results may have underestimated the difference between linear and circRNAs levels. The correlation between the regulation of linear and circRNAs would be even lower if we were able to separate the read counts. The EdgeR R package (version 3.6.8) was used for differential expression analysis^51^. This package uses the Trimmed Mean of M-values (TMM) normalization method to remove systematic technical effects that occur in the data to minimize the impact of technical bias on differential expression analysis results^52^. Moreover, the empirical Bayes method used in the package enables gene-specific variation estimates even when the number of replicate samples is very small. This method has been demonstrated in experiments with only two replicates^53^, and thus is particularly appropriate for our study. For differential expression analysis of circRNAs, back-splicing read counts of circRNAs were added to the bottom of gene count list as new genes for the normalization purpose. The cutoffs for log2 fold change (log2FC) and FDR were |log2FC| > 1 and FDR < 0.01.

### Evaluate circRNA candidates by paired end information

To evaluate circRNA candidates by paired end sequencing information, corresponding mates of paired end reads were initially extracted for back-splice mapped mates. Then, fragments from the 5′ ends of linear transcripts of circRNAs with length 100 nt were copied to the 3′ end of these linear transcripts. These mates were then mapped to the modified linear sequences using Tophat2 with up to 2 mismatches. PCMM values were calculated as the number of reads both mates are mappable/the number of reads with back-splice mapped mate.

### Compare expression levels between circRNAs and linear transcripts

To compare the relative expression levels between exons with and without circRNA candidates, DEPTH tool from Samtools package (version 0.1.19–44428 cd) was used to report read depths for genes with circRNA candidates^54^. The mean value of read depths from an exon was used as the read depth of the exon. EWC/ENC value was calculated as the mean depth of exons with circRNAs/without circRNAs.

### RT-PCR

To validate circRNA species, 0.5 ug of total RNA was reverse transcribed in a 30 μl reaction using AccuScript Hi-Fi RT kit with random hexamers according to manufactures protocol (#200820, Agilent Technologies). The resultant cDNA was diluted 4-fold in RNase- and DNase-free water and approximately 14 ng was used as template for each qPCR reaction. qPCR was performed in technical triplicates for each amplicon using SsoAdvanced Universal SYBR^®^ Green Supermix (Bio-Rad). qPCR reactions were conducted on a Bio-Rad CFX384 instrument and relative expression levels were obtained using cycle threshold (Ct) values obtained by instrument software. All Ct values ≥31 were considered as background and discarded from further analysis. Triplicate C(t) values were averaged and normalized to U6 snRNA. Fold-changes were calculated using the ΔΔC(t) method, where: Δ = C(t)circRNA - C(t)U6 snRNA, and ΔΔC(t) = ΔC(t)DKO or DLD − ΔC(t)DKs, and FC = 2^ΔΔC(t). Analysis was performed on three independent cell and exosomal samples. Forward (F) and reverse (R) primers used in qPCR analysis were designed against head-to-tail junctions of putative circRNA products as follows: FAT1- (F) ACGCCAGAGCCATCTCTAAT, (R) GCAATGGGGAGACATTTGGC; HIPK3- (F) ATGGCCTCACAAGTCTTGGT, (R) TGGCCGACCCAAAGTCTATT; ARHGAP5- (F) TGATCTTGAAGATGTTTCTGCACAG, (R) CATCTAACTCCTGGTCAGAAGTG; MAN1A2- (F) TTCGAGCTGATCATGAGAAGG, (R) GCAAGTAGGCCTCCAATAAA; RHOBTB3- (F) TAAAGGCTGAAGCGTCACATTAT, (R) CTCGATTACATTTGAAACATCCCCA; RTN4- (F) CAACTAAGAAGAGGCGCCTG, (R) AGACTGGAGTGGTGTTTGGT; SMARCA5- (F) GGCTTGTGGATCAGAATCTGAACA, (R) TCTCTATAGTCTTCTCCTTCGAAGT. All primer sequences are 5′ to 3′ (Table S5). Table S6 includes primer details and sequence information for the linear RNA species. The primer sequences were blasted against the NCBI human genomic + transcript database to ensure specific amplification of the intended targets. Moreover, the melt curves showed that each primer set only had one specific peak, suggesting that the amplicon was specific and no other secondary targets were being amplified.

## Additional Information

**How to cite this article**: Dou, Y. *et al*. Circular RNAs are down-regulated in *KRAS* mutant colon cancer cells and can be transferred to exosomes. *Sci. Rep.* **6**, 37982; doi: 10.1038/srep37982 (2016).

**Publisher's note:** Springer Nature remains neutral with regard to jurisdictional claims in published maps and institutional affiliations.

## Supplementary Material

Supplementary Figures and Tables

Supplementary Table S3

## Figures and Tables

**Table 1:** Identification of circRNA candidates in the three cell lines.

Sample	DKs-8.1	DKs-8.2	DLD-1.1	DLD-1.2	DKO-1.1	DKO-1.2
Paired-end reads	88746986	111215656	100025762	97930613	107392430	91024681
Back-splice reads	65319	80701	47941	44717	40657	37403
circRNA candidates	11061	13565	8771	8182	7348	6827
High quality candidates	932	1211	651	571	488	428
Host genes	676	866	509	455	392	342
Genes with more than one high quality candidates	158	192	87	72	57	53

**Table 2:** Top10 most abundant circRNAs in distinct genes in DKs-8 and their differential expression results.

Candidates	Gene	circRNA comparisons			
		DLD-1/DKs-8		DKO-1/DKs-8	
		Log2FC	FDR	Log2FC	FDR
chr4:187627717-187630999	FAT1	−2.71	0	−2.41	2.01E-267
chr11:33307959-33309057	HIPK3	−0.81	1.26E-22	−1.07	1.40E-35
chr14:32559708-32563592	ARHGAP5	−1.12	2.37E-34	−1.10	2.67E-32
chr1:117944808-117963271	MAN1A2	−1.27	1.52E-25	−1.59	9.14E-36
chr5:95091100-95099324	RHOBTB3	−1.00	1.97E-16	−1.54	4.30E-32
chr2:55209651-55214834	RTN4	−1.81	2.69E-33	−2.04	1.93E-39
chr17:20107646-20109225	SPECC1	−0.45	5.15E-04	−0.94	4.83E-12
chr4:144464662-144465125	SMARCA5	−0.81	2.43E-09	−1.07	1.32E-14
chr4:25789846-25804084	SEL1L3	−0.07	9.05E-01	−0.17	3.72E-01
chr20:30954187-30956926	ASXL1	−0.50	9.78E-04	−0.66	1.68E-05

**Table 3:** Top10 most abundant circRNAs in distinct genes in DKs-8 exosomes.

Candidates	Gene	DKs-8.1		DKs-8.2		DKs-8.3	
		Back-splice reads	PCMM	Back-splice reads	PCMM	Back-splice reads	PCMM
chr5:95091100-95099324	RHOBTB3	81	95.1%	141	92.2%	89	95.5%
chr11:33307959-33309057	HIPK3	75	81.3%	97	88.7%	51	90.2%
chr4:187627717-187630999	FAT1	69	88.4%	78	94.9%	60	90.0%
chr4:144464662-144465125	SMARCA5	44	97.7%	95	95.8%	61	88.5%
chr1:117944808-117963271	MAN1A2	36	83.3%	65	95.4%	37	94.6%
chr20:30954187-30956926	ASXL1	28	85.7%	60	85.0%	33	90.9%
chr12:120592774-120593523	MIR4498	40	92.5%	44	97.7%	28	89.3%
chr2:55209651-55214834	RTN4	28	85.7%	43	88.4%	35	100.0%
chr9:138773479-138774924	CAMSAP1	21	95.2%	36	91.7%	34	82.4%
chrM:2003-2226	MT-RNR2	67	92.5%	18	90.9%	3	100.0%
