# Supplementary material for: Circular RNAs are down-regulated in KRAS mutant colon cancer cells and can be transferred to exosomes
Source: Sci Rep. 2016 Nov 28;6:37982. doi: 10.1038/srep37982 (PMC5125100; doi:10.1038/srep37982)
Supplement: Supplementary Figures and Tables [file srep37982-s1.pdf]

Circular RNAs are down-regulated in KRAS mutant colon cancer cells and can be transferred to exosomes

Yongchao Dou[1], Diana J. Cha[2], Jeffrey L. Franklin[3,4], James N. Higginbotham[3,4], Dennis K. Jeppesen[4], Alissa M. Weaver[4,5,6], Nripesh Prasad[7], Shawn Levy[7], Robert J. Coffey[3,4], James G. Patton[2] and Bing Zhang[1]*

[1] Department of Biomedical Informatics, Vanderbilt University, Nashville, Tennessee 37232, USA
[2] Department of Biological Sciences, Vanderbilt University, Nashville, Tennessee 37232, USA
[3] Department of Cell and Developmental Biology, Vanderbilt University, Nashville, Tennessee 37232, USA
[4] Department of Medicine, Vanderbilt University, Nashville, Tennessee 37232, USA
[5] Department of Cancer Biology, Vanderbilt University Medical Center, Nashville, Tennessee 37232, USA
[6] Department of Pathology, Microbiology and Immunology, Vanderbilt University Medical Center, Nashville, Tennessee 37232, USA
[7] HudsonAlpha Institute for Biotechnology[5], Huntsville, Alabama 35806, USA

*To whom correspondence should be addressed:

Bing Zhang, bing.zhang@vanderbilt.edu

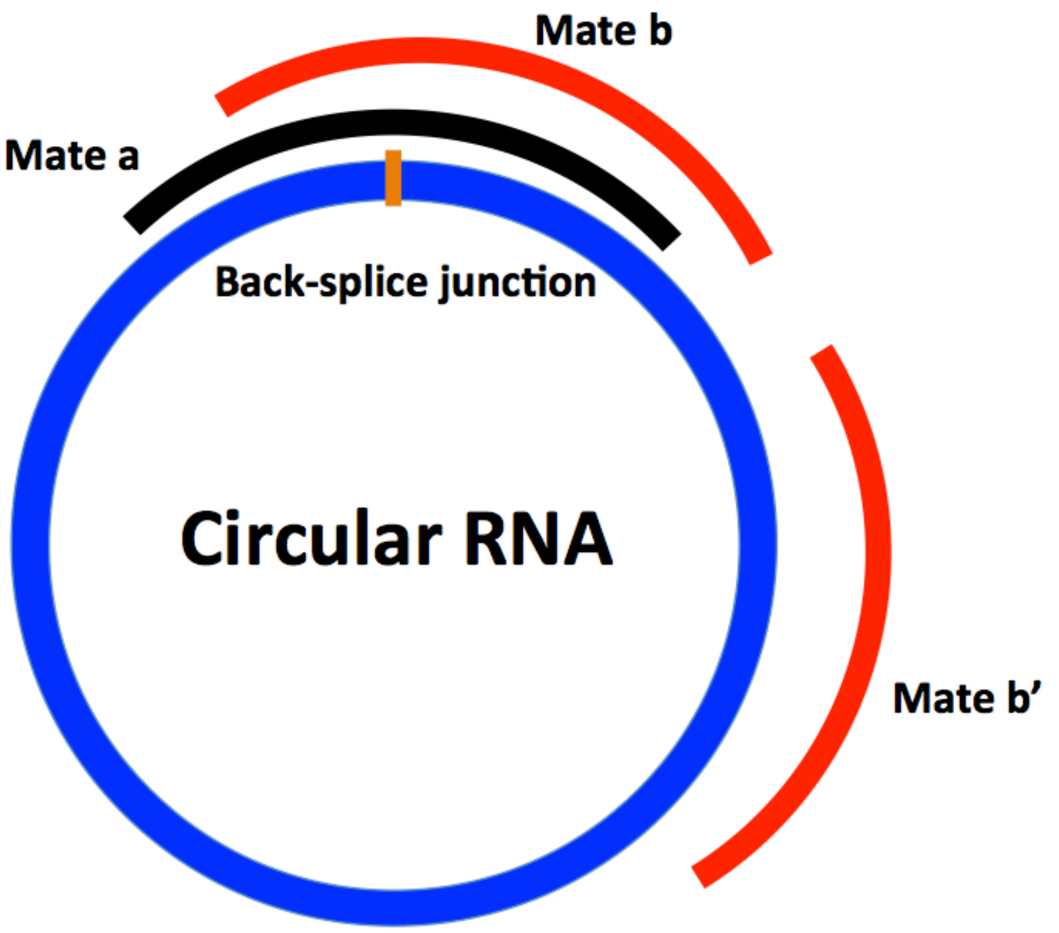

Figure S1. Mapping of paired end reads to circRNAs.

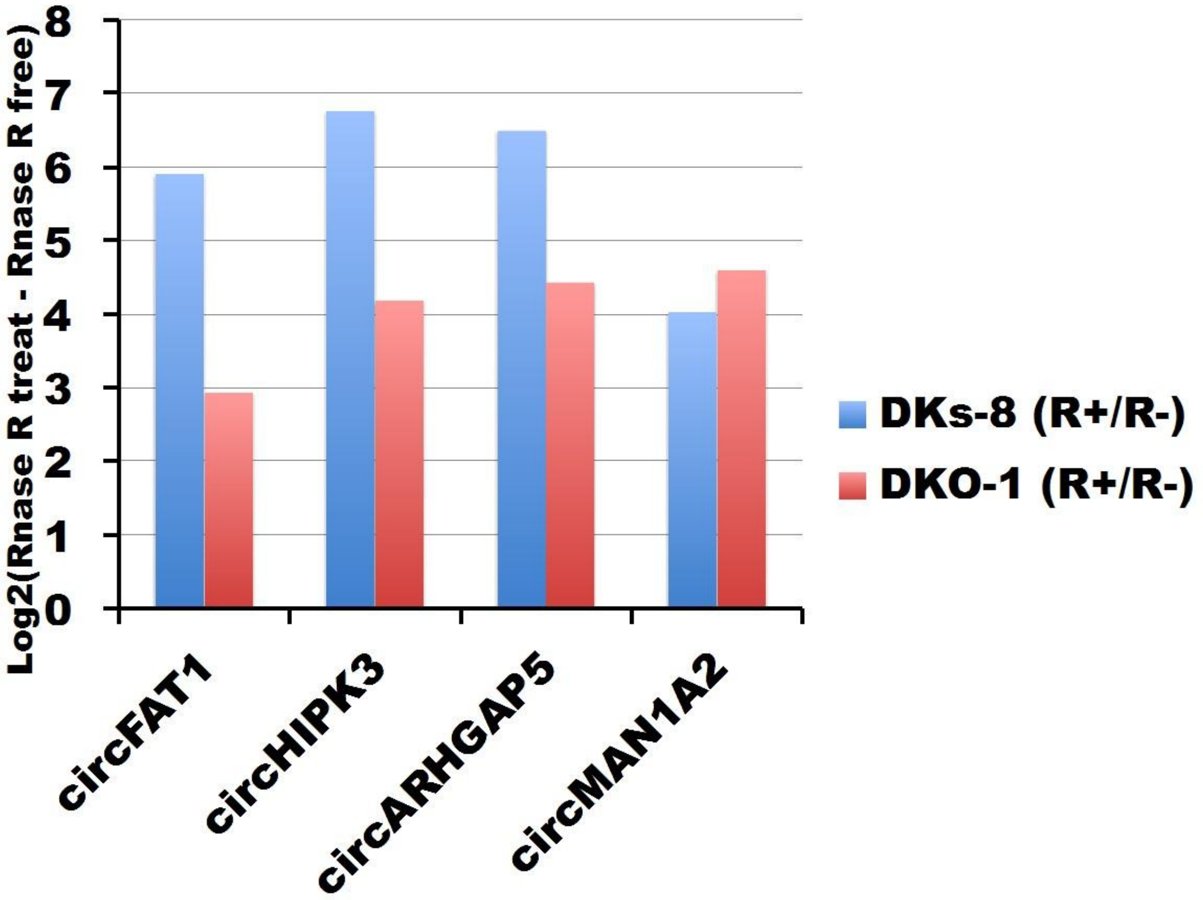

Figure S2. Enrichment of circRNAs after Rnase R treatment.

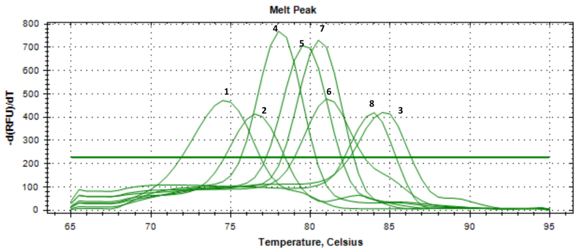

Peak	Target	Sample	Melt Temperature	Peak Height
1	circFAT1	DKO_1	74.50	471.62
2	circHIPK3	DKO_1	76.50	413.84
3	circARHGAP5	DKO_1	84.50	420.24
4	circMAN1A2	DKO_1	78.00	769.23
5	circRTN4	DKO_1	79.50	706.81
6	circRHOBTB3	DKO_1	81.00	478.73
7	circSMARCA1	DKO_1	80.50	730.54
8	GAPDH	DKO_1	84.00	419.34

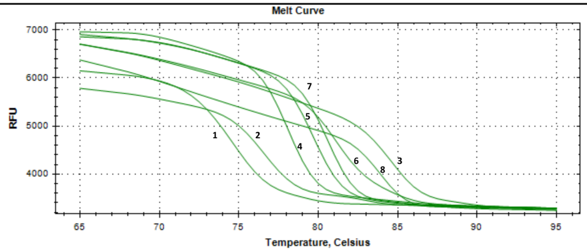

Peak	Target	Sample	Melt Temperature	Peak Height
1	circFAT1	DKO_1	74.50	471.62
2	circHIPK3	DKO_1	76.50	413.84
3	circARHGAP5	DKO_1	84.50	420.24
4	circMAN1A2	DKO_1	78.00	769.23
5	circRTN4	DKO_1	79.50	706.81
6	circRHOBTB3	DKO_1	81.00	478.73
7	circSMARCA1	DKO_1	80.50	730.54
8	GAPDH	DKO_1	84.00	419.34

Figure S3: melt curves for each set of primer

Table S1: blast results of back-splicing mates

Sample	back-splicing mates #	No hits #	High quality #
DKs-8.cell.1	75163	75039(99.8)%	0(0.0)%
DKs-8.cell.2	92136	91945(99.8)%	0(0.0)%
DLD-1.cell.1	54855	54703(99.7)%	0(0.0)%
DLD-1.cell.2	50945	50828(99.8)%	0(0.0)%
DKO-1.cell.1	46282	46110(99.6)%	0(0.0)%
DKO-1.cell.2	42508	42384(99.7)%	0(0.0)%
DKs-8.exo.1	14290	14198(99.4)%	0(0.0)%
DKs-8.exo.2	25830	25084(97.1)%	1(0.0)%
DKs-8.exo.3	21101	20030(94.9)%	3(0.0)%
DLD-1.exo.1	2240	2172(97.0)%	0(0.0)%
DLD-1.exo.2	17088	16281(95.3)%	6(0.0)%
DLD-1.exo.3	35236	34565(98.1)%	4(0.0)%
DKO-1.exo.1	23693	23559(99.4)%	0(0.0)%
DKO-1.exo.2	8131	7468(91.8)%	1(0.0)%
DKO-1.exo.3	8911	8488(95.3)%	0(0.0)%

Table S2: Back-splicing mates mapped to bovine genome

Sample	back-splicing mates #	back-splicing mapped	Linear mapped
DKs-8.cell.1	75163	1614(2.1%)	5(0.0%)
DKs-8.cell.2	92136	1997(2.2%)	5(0.0%)
DLD-1.cell.1	54855	1017(1.9%)	5(0.0%)
DLD-1.cell.2	50945	989(1.9%)	9(0.0%)
DKO-1.cell.1	46282	892(1.9%)	3(0.0%)
DKO-1.cell.2	42508	829(2.0%)	5(0.0%)
DKs-8.exo.1	14290	287(2.0%)	1(0.0%)
DKs-8.exo.2	25830	510(2.0%)	4(0.0%)
DKs-8.exo.3	21101	361(1.7%)	4(0.0%)
DLD-1.exo.1	2240	18(0.8%)	2(0.1%)
DLD-1.exo.2	17088	326(1.9%)	3(0.0%)
DLD-1.exo.3	35236	626(1.8%)	6(0.0%)
DKO-1.exo.1	23693	528(2.2%)	4(0.0%)
DKO-1.exo.2	8131	137(1.7%)	0(0.0%)
DKO-1.exo.3	8911	129(1.4%)	1(0.0%)

Table S4: Identification of circRNA candidates in exosomes

Sample	Paired-end reads	Back-splice reads	circRNA candidates	high quality candidates	Host gene	Genes with more than one high quality candidates
DKS8.exo.1	120350227	13001	2651	84	72	3
DKS8.exo.2	113418948	23263	4392	195	166	8
DKS8.exo.3	115990041	19121	3308	119	100	3
DKO1.exo.1	123041690	2074	527	2	2	0
DKO1.exo.2	114041294	15700	2801	87	68	3
DKO1.exo.3	109044100	32016	6088	319	271	27
DLD1.exo.1	120736524	21476	4945	218	195	12
DLD1.exo.2	119110502	7563	1391	31	19	1
DLD1.exo.3	120405815	8327	1429	37	27	2

Table S5: qRT-PCR primers used for circRNAs

Candidates	Host gene	Exon/intron		F primer	R Primer
Chr4: 187627717-187630999	FAT1	(as) Exon 2-1, intron 1-2	CACAGCCGAGTACTACTCCTGCGGTCTTCTCTACCCCTCTAGGCTATGAGGTAATCTCTACCGAGACCGCAACCACAAAAGTTTTATCCACTTCTCTGTC / TAAGGGCTGTCAATTCGTTACCCCTCTGTAAACCGAAACGAGGACGAAGACGAGGAAGAGAAGGTTGTAAAACCTCTGTCACTACCGTCGGTTGCTGAAC	ACGCCAGAGCCATCTCTAAT	GCAATGGGGAGACATTTGGC
Chr11:33307959-33309057	HIPK3	Exon 2	GTTGACGAACCGAGATGAAACTCAAAGAAGTGTGATGTTTTCCGTGAACTGACTCAAACTATTTGTATACCACCCATCTGGTTCTGAACACTCCGGTATG / GACATCATGGCTCTAACATCTATACAACTTGTTTGTCAGAAACTATGTACTGACCGGCTGGGTTTCAGATAATGAAATTGGGACATTCCGACGGCTTGTC	ATGGCCTCACAAGTCTTGGT	TGGCCGACCCAAAGTCTATT
Chr14: 32559708-32563592	ARHGAP5	Exon 2	TAGAGTTTTATGTCTAGGGTTTAAAGTCTTGACATAAACCAAGAAACATAAAGGGAGTAAAGACACGTCTTTGTAGAAGTTCTAGTATCCCTAGTAGAAG / GAAAAATAGAAGAAAGTGAAACACCCAAAAGAAGAAGTAGAAGAATAGTAGATTGAGGACCAGTCTTCACTAACGTCCTAATAGTTATGGTGGAAAGGTT	TGATCTTGAAGATGTTTCTGCACAG	CATCTAACTCCTGGTCAGAAGTG
Chr1: 117944808-117963271	MAN1A2	Exon 2-5, intron 2-5	AGCTTAAAGGAGAGAACTGAAAGAATTAAAAAGAAAACGAAGAAGGTTCCGGAAGAGTACTAGTCGAGCTTAAAATAAAGAGTCTGCAAGAAGGAGAAGG / GAGGAGAGGACTATCCATTATACGACGTTCATCCGGAGGTTATTTAGCTTACAACTGAAGTTTGTGTCTGTGGAGACTTAAGTGTGACTTTAGTTCCAAC	TTCGAGCTGATCATGAGAAGG	GCAAGTAGGCCTCCAATAAA
Chr5:95091100-95099324	RHOBTB3	Exon 6-7, intron 6-7	CCCAACATTTTATGGTGCAGGTGTGTGACCGTCGTCTTGTCGTTCAATAAATTCAGTCTCAATATTACACTGCGAAGTCGGAAATTCTGTCCGTAAAAAA / GAAAGGACCACAAAAATTTTACGTAAATTGAAAGAGCTAATGTAAACTTTGTAGGGGTCTTAGAAAGTTGAAGAAGGACTATAGAAGAAGGTTAAGAAGG	TAAAGGCTGAAGCGTCACATTAT	CTCGATTACATTTGAAACATCCCCA
Chr2: 55209651-55214834	RTN4	(as) exon 5- 4, intron 5-4	CCAAGTCTTCATGTCATTAAGACGAGAACCAGTACACTTGACGTGCTATTTCCTTGAGTCCGCGGAGAAGAATCAACTACTAAATCAACTAAGAGACTTC / AACAACTGGAGGACATGACCTCTCTGTAATTCTTCTGACCTCACCACAAACCACGGTCGGATAAGGACGACGAAAGTAACTGTCATAAGTCGTAACACTC	CAACTAAGAAGAGGCGCCTG	AGACTGGAGTGGTGTTTGGT
Chr4: 144464662-144465125	SMARCA5	Exon 15-16/ intron 15-16	AGTGAAAGGAAACTTCGTTTGTGTACACAACGAGGTACAGATTAGTAAACTTCGTAAAGTAGAAAGGGTTAAAACAAGTCTAAGACTAGGTGTTCGGAGG / GAAAACAAAAAGAGATATCAGAAGAGGAAGCTTCAATATTTGTGAACTGAGACATAGGTAACATTTCAAAGATTCACTTGAAAGCGGGTAGAACCTCTCG	GGCTTGTGGATCAGAATCTGAACA	TCTCTATAGTCTTCTCCTTCGAAGT

Table S6: qRT-PCR primers used for the host genes of selected circRNAs

linear RNAs	F primer	R primer	Exon- exon junction	Amplicon size
FAT1	GCTCTGGACAAGGACAAAGG	AGCCCAAGACAGGATCAATCA	9-10	97
HIPK3	CCCCTGCAAGAGACCGAATA	GCCATCCACCGTATCACAAC	14-15	67
ARHGAP5	CTGCAATCACTTCTGACCAGG	TCTACGTGTTGGTGGATTGAAG	2-3	115
MAN1A2	TGCACGTACTTGTCATGAGTC	ATACTTTTCAGCCTGCCGGA	10-11	112
RHOBTB3	CCACCTCAACTTGAACAACCA	GGCAGCAGAACAGCAAGTTA	5-6	94
RTN4	CATCTGAGCCTGTGATACGC	ATCCTCTTGACCAGCCGAAA	2-3	86
SMARCA5	TCAATCTTGCGACTGCTGATG	TCTGACTGTCTTAGTCTGCCC	13-14	113
